# Measurement of breastfeeding initiation: Ethiopian mothers’ perception about survey questions assessing early initiation of breastfeeding

**DOI:** 10.1186/1746-4358-9-13

**Published:** 2014-08-25

**Authors:** Mihretab Melesse Salasibew, Suzanne Filteau, Tanya Marchant

**Affiliations:** 1 London School of Hygiene and Tropical Medicine, Keppel Street, London WC1E 7HT, UK

**Keywords:** Early initiation, Breastfeeding, Infant feeding, Breast milk, Essential interventions, Saving newborn lives

## Abstract

**Background:**

Although breastfeeding is almost universal in Ethiopia, only 52% newborns benefited from early initiation in 2011. Early initiation is one of the recommended interventions for saving newborn lives but its potential seems not yet realized for Ethiopian newborns and there is a need for continued efforts to increase coverage. To do so, it is also relevant to focus on consistent and accurate reporting of coverage in early initiation.

WHO recommends the question “how long after birth did you first put [name] to the breast?” in order to assess coverage in early initiation. It is designed to measure the time after birth when the mother attempted to initiate breastfeeding regardless of whether breast milk had arrived or not. However, it is unclear how mothers perceive this question and what their responses of time refer to. In this study, we assessed Ethiopian mothers’ perception about the question assessing early initiation.

**Methods:**

Cognitive interviews were conducted between April and May 2013 with eligible mothers in Basona and Debrebirhan woredas (districts), 120 km away from Addis Ababa, Ethiopia.

**Results:**

A total of 49 mothers, most from Basona (n = 36) and the rest from Debrebirhan woredas (n = 13) were interviewed. No probes or follow on questions were required for mothers to understand what the WHO recommended question was about. However, further probing was needed to ascertain what maternal responses of time refer to. Accordingly, mothers’ response about the timing of early initiation was related to the first time the newborn received breast milk rather than their first attempt to initiate breastfeeding. In addition, considerable probing was required to approximate and code responses of time based on the WHO coding format because some mothers were unable to assess time in minutes or hours.

**Conclusion:**

The existing question is not adequate to identify intended attempts of mothers to initiate breastfeeding. We recommend revising the question as “how long after birth did you first put [name] to the breast even if your breast milk did not arrive yet?” Standard probes or follow on questions are required to avoid subjective interpretation of the indicator.

## Background

Globally, a marked 41% reduction has been recorded in under-five mortality: from 87 deaths per 1,000 live births in 1990 to 51 in 2011. Sub-Saharan Africa, still with the highest mortality rates in the world, achieved double reduction from 1.5 per cent a year in 1990 – 2000 to 3.1 per cent a year in 2000 – 2011. However, most of the reduction has been in deaths of older infants and toddlers so the proportion of all under-five deaths that occurred in the neonatal period (first 28 days of life) worldwide increased from about 36 per cent in 1990 to 43 per cent in 2011 [1]. Ethiopia is among seven high-mortality countries (Bangladesh, Ethiopia, Liberia, Malawi, Nepal, United Republic of Tanzania and Timor-Leste) which have already achieved the fourth millennium development goal with 67% reduction in under-five mortality between 1990 and 2012 although the proportion of neonatal deaths still remains high [2].

Interventions immediately after birth such as breastfeeding, thermal care to prevent hypothermia (through immediate drying, warming, skin-to-skin contact and delayed bathing) and hygienic cord and skin care have been recommended to save newborn lives [3]. It was estimated that if coverage was universal, exclusive breastfeeding, thermal care and cord care could save up to 13%, 2% and 4% of all under-five deaths respectively [4]. Further, studies have estimated that up to 16% of all neonatal deaths could be saved if all infants were breastfed within the first day of life, and 22% if breastfed within the first hour after birth (also referred to as ‘early initiation’) [5]. Although breastfeeding is almost universal in Ethiopia, with over 98% of all children ever breastfed, the 2011 Demographic and Health Survey (DHS) estimated that only 52% of Ethiopian newborns benefited from early initiation of breastfeeding which is far from the government’s own target of 92% [6]. As such, the potential of early initiation of breastfeeding to save newborn Ethiopian lives has not yet been realized and there is a need for continued efforts to increase coverage.

To do so, it is also relevant to focus on consistent and accurate reporting about coverage in early initiation since these are needed to inform policy makers and programme managers about successes and failures of actions, and to make comparison of results across time and place. In 1991, the World Health Organization (WHO) recommended standard questions and indicators for the assessment of early initiation of breastfeeding in household surveys (Table 1). The main purpose of setting standards was to have a common set of measurement tools in order to assess practices and evaluate promotional programs [7]. These standards were revised in 2007 following changes in infant feeding recommendations [8]. In 2010, WHO published a further guideline on how breastfeeding indicators should be measured. These include detailed guidance on the text description of survey questions, sampling strategy, choice and interpretation of indicators [9].

**Table 1:** Standards recommended for assessing coverage in early initiation of breastfeeding

WHO standard definition	breastfeeding initiation within an hour after birth.	
Indicator	Children born in the last 24 months who were put to the breast within one hour of birth divided by children born in the last 24 months.	
Survey question	How long after birth did you first put (NAME) to the breast?	
Coding format	If mother reports she put the infant to the breast immediately after birth, circle ’000’ For ‘Immediately’.	IMMEDIATELY.................000
	If less than 1 hour, circle ‘1’ for hours ANDRECORD‘00’ hours.	HOURS.............................1 |___|___|
	If less than 24 hours, circle ‘1’ and record number of completed hours, from 01 to 23.	HOURS.............................1 |___|___|
	Otherwise, circle ‘2’ and record number of completed days.	DAYS.............................2 |___|___|

Eligible mothers to be asked the question assessing early initiation of breastfeeding are those between 15 to 49 years old and ever breastfed a child born two years preceding the survey. WHO also provides the following guidance about the questions assessing ‘ever breastfeeding’ and ‘initiation of breastfeeding’.

*Child ever breastfed*

For this question it does not matter how long the respondent breastfed the child, only whether or not she ever gave the child the breast (even if the baby died very young). It does not matter whether or not the mother’s milk had arrived at the time she gave the child the breast.

*Initiation of breastfeeding*

*This question asks about when the child was first put to the breast. For this question, it also does not matter whether or not the mother’s milk had arrived at the time of first putting the child to the breast.*[9]*, p.18*

Accordingly, the indicator for early initiation is designed to measure the length of time after birth when the mother attempted to initiate breastfeeding regardless of whether breast milk had arrived or not. A mother putting her baby directly to her breast and trying to get the baby’s mouth to latch to the nipple is an attempt to initiate breastfeeding. Similarly, having the newborn baby placed on mother’s chest with skin-to-skin contact could also be regarded as attempting to initiate breastfeeding. Because, studies have shown that newborn babies placed on mothers’ chest with skin-to-skin contact would naturally make predictable movements or crawl towards the breast and are likely to initiate and successfully continue breastfeeding afterwards [10-14]. Such evidence formed the basis for the WHO recommendation of an arbitrary but practical minimum timing for mother-baby skin-to-skin contact to start within at most half an hour of birth and to continue for at least 30 minutes [15].

However, it is not clear how mothers participating in breastfeeding surveys perceive the early initiation question and what their responses of time refer to. Such understanding is necessary in order to ensure accuracy in reported coverage figures about early initiation. In this study, we investigated how Ethiopian mothers perceive the early initiation question and whether their responses of time refer to their first attempt to initiate breastfeeding either by putting the baby to the breast or placing baby on mother’s chest with skin-to-skin contact even if baby didn’t manage to get breast milk.

## Methods

### Study design

We used a qualitative study design for in-depth understanding about how mothers perceive the wording or text description of the question assessing early initiation of breastfeeding.

### Study setting

The study was conducted in two woredas (districts) namely; Basona (predominantly rural) and Debrebirhan (predominantly urban) woredas. These woredas are located in Amhara regional state and 120 km away from Addis Ababa, the capital city of Ethiopia.

### Study participants

Women residents either in Basona and Debrebirhan woredas, aged 15–49 years, who had at least one live birth in the last two years and with the most recent child ever breastfed were participants of the study as per WHO criteria for inclusion and eligibility to be asked the question assessing early initiation of breastfeeding [9]. Health extension workers, who provide integrated preventative care services within communities in Ethiopia, assisted in the identification and selection process. These workers have a list of all 15–49 years old mothers in their catchment area, which we used to select study participants based on the eligibility criteria.

### Data collection

We used cognitive interviewing technique with verbal probing to interview eligible mothers over a 6 weeks period between April and May 2013. The principal investigator conducted all interviews using the local language Amharic. The number of interviews conducted was determined by reaching the saturation point i.e. when there are no more new emerging views coming from mothers. In Basona woreda, interviews were held in a room provided by the local government administrative unit whereas in Debrebirhan they were held in residential homes of eligible mothers. A total of 49 mothers, most from Basona (n = 36) and the rest from Debrebirhan woredas (n = 13), were interviewed in the study. All interviews were tape-recorded after receiving informed written consent or thumb prints in order to do so. Prior to each interview, we completed a form to collect demographic characteristics of eligible mothers including their woreda (Basona or Debrebirhan), level of education (illiterate or ≥ primary level education), place of delivery (home or hospital), time since last birth (<6 or 6–24 months) and parity (first time mother or with more than one child).

To identify attempts of breastfeeding initiation either by putting the baby directly to the breast or placing the baby on mother’s chest with skin-to-skin contact, we asked mothers either of the following two questions and assessed their perception.

**Q1: “how long after birth did you first put [name] to the breast?”** (Standard WHO question).

Of the total 49, we interviewed 11 eligible mothers using this question when saturation point was reached.

**Q2: “how long after birth did you first put [name] in contact with you on your chest?”** (Revised WHO question in order to detect attempts to initiate breastfeeding through skin-to-skin contact).

Of the total 49, we interviewed 38 mothers using this question; 6 in Debrebirhan and 32 in Basona woreda. More mothers were interviewed using this question (Q2) to get to saturation point especially in the rural woreda Basona.

During the cognitive interview process, we first read the questions either Q1 or Q2 and asked mothers the first thing they thought upon hearing the questions before they responded with a time for breastfeeding initiation. We then used further probes to determine whether or not their responses of time refer to the time between birth and the mother attempting to initiate breastfeeding even if baby actually didn’t start getting mothers’ milk. (See Additional file 1: Topic guide).

### Data analysis

All interviews were transcribed verbatim directly from Amharic audios into English transcripts initially by an academic from Debrebirhan University and then by the principal investigator. Data was analysed deductively using the frame work analysis approach. Using qualitative data analysis software package NVIVO version 10, each transcript was carefully screened and coded. These codes were in turn grouped in to major themes representing similar views or perception about the questions.

### Ethical issues

The study received ethical approval from Observational/Interventions Research Ethics Committee at the London School of Hygiene and Tropical Medicine in October 2012 and in Ethiopia from the National Research Ethics Committee under the Ministry of Science and Technology in April 2013. Further support letters were also obtained from Ethiopian Ministry of Health, Debrebirhan University and the health bureaus in Basona and Debrebirhan woredas. All eligible mothers gave written informed consent or thumb print prior to interviews.

## Results

A total of 49 mothers, most from Basona (n = 36) and the rest from Debrebirhan woredas (n = 13), were interviewed in the study (Table 2). Of those interviewed in Basona woreda, 16 and 20 were from rural villages called Ametsegna and Kachamba respectively whereas in Debrebirhan, all mothers interviewed were from Kebele 09 (sub-district).

**Table 2:** Characteristics of mothers interviewed

**Characteristic**	**N (%)**
Woreda	
Basona	36 (74)
Debrebirhan	13 (26)
Educational level	
Illiterate	19 (39)
≥ primary level education	30 (61)
Place of delivery	
Home	27 (55)
Hospital	22 (45)
Parity	
Primiparous	10 (20)
Multiparous	39 (80)
Time since last delivery	
< 6 months	12 (24)
6-24 months	37 (76)

### Immediate perception up-on hearing the questions

1.1 Q1: “how long after birth did you first put [name] to the breast?”

We asked 11 mothers this question and the first thing these mothers thought upon hearing the question was about their practice of breastfeeding initiation and tried to recall the time it took them to do so immediately after birth.

“Your question is about what I fed my baby immediately after birth? …you asked if she breastfed immediately or waited for a while. . . . I don’t think it took 2 hrs, because she is my first daughter [other children are boys] and I was eager to have her with me soon after birth. When I asked them [traditional birth attendants] to give me my daughter, they told me to wait a bit until they give her bath and do other things. When they gave me about 2 hrs later, I gave her my breast and she got milk soon” [Mother of 3, illiterate, home delivery, Basona woreda].

“ . . . well, you asked me about breastfeeding and it is one and half hour later that I started breastfeeding and she got milk soon” [First time mother, literate, with 2 weeks old baby born in hospital, Debrebirhan woreda].

No probes or follow on questions were required for these mothers to understand what the question was about. However, probes were needed to ascertain whether or not their responses of time referred to the time after birth when they attempted to initiate breastfeeding even if baby didn’t actually get their breast milk yet.

1.2 Q2: “how long after birth did you first put [name] in contact with you on your chest?”

Thirty eight mothers were asked this question. The majority thought the question was about breastfeeding and didn’t need further probes or follow on questions to understand what the question was about. However, 5 mothers asked for further clarification and needed probing before they were able to give their responses of time as illustrated below:

“What do you mean when you say on my chest?” [Mother of 3, literate, hospital delivery, Basona woreda].

“ . . . do you mean hugging her while I was at the hospital?” [Mother of 2, literate, hospital delivery, Basona woreda].

Others (n = 7) perceived the question differently and the first thing they thought upon hearing the question was about the time when they started hugging or holding the baby on their chest.

“After 6 months . . . I mean she becomes stronger after 6 months and even she tries to stand when I hold her in my chest” [illiterate, 2 children, Basona woreda].

“After 5 months . . . you asked me how long after birth did I put my baby in to my chest and that is after 5 months” [Literate, 3 children, Basona woreda].

Even after repeating the question and further probes, a mother [Mother of 3, illiterate, home delivery and Basona woreda] still did not understand the question asked was about breastfeeding initiation as shown in the extract below:

MS: how long after birth did you first put [name] in contact with you on your chest?

Mother: it is after 3 days or so that the baby becomes slightly stronger for me to hug and hold her in upright position in my chest. We can’t hug the baby soon after her birth and sit down…rather you wrap the baby soon after birth and get her to sleep or rest . . .

MS: Ok thanks, can I ask if you understood my question please?

Mother: you asked me about the circumstances around [name’s] birth…

MS: Ok, may be let me repeat the question . . . how long after birth did you first put [name] in contact with you on your chest?

*Mother: I said it is not possible to hug the baby soon after birth, which means it would be on the 3*^*rd*^*or 4*^*th*^* day after birth that I hug my baby.*

This particular mother was able to give relevant responses of time for breastfeeding initiation once the phrase “start breastfeeding” was included in the question.

MS: Ok, how long after birth did you start breastfeeding [name] then?

Mother: we don’t give breast milk soon after birth. We give the baby slightly warm water with some sugar using a spoon. This continues until the breast milk bursts and the mother gets ready to feed. We don’t give butter these days either . . . …, she was born at 2 am and she started breastfeeding around 12 pm noon [10 hrs after birth].

Therefore, revising the early initiation question as Q2 (how long after birth did you first put [name] in contact with you on your chest?) confused some mothers resulting in unintended responses of time for breastfeeding initiation and considerable probing was required. Although we revised the standard WHO question to detect attempts of breastfeeding initiation by placing baby on mother’s chest with skin-to-skin contact, none of the mothers we interviewed reported having skin-to-skin contact with their babies immediately after birth. Instead during home deliveries, babies were given bath immediately after birth, dried and wrapped up in a towel before attempting to initiate breastfeeding.

“When the baby came out, she (traditional birth attendant) cut the cord using clean blade and tied it. After that, she wrapped the baby in a towel and gave me to breastfeed . . . ” [First time mother, literate, Basona woreda].

Therefore, maternal responses of time to both questions Q1 & Q2 refer to the first time the newborn received breast milk rather than their first attempt to initiate breastfeeding. In fact, some mothers did not even consider colostrum as part of breast milk and they reported the time after birth when their milk ‘bursts’ or ‘came in’: in other words, when full lactation was established. For example, a first time mother, illiterate and from Basona woreda reported that it took her up to 3 days to initiate breastfeeding although colostrum was available to the baby during her earlier attempts to initiate breastfeeding.

MS: how long after birth did you first put [name] in contact with you on your chest?

Mother: . . . I started feeding 3 days later because the milk was not coming until 3 days.

MS: ok, have you actually tried to breastfeed your baby soon after birth even if the milk was not coming?

Mother: yes, I tried but no success. The milk came 3 days later. I gave her cow milk for the first 3 days until the breast milk came in . . .

### Mothers’ understanding of time

The indicator for early initiation is time-dependent and the WHO coding format (Table 1) [9] is designed to capture responses of time in minutes, hours or days. However, some mothers we interviewed found it difficult to assess the time as such.

“I don’t know time . . . ? It is difficult to say . . . ” [Mother of 2, illiterate, home delivery, Basona woreda].

When mothers were able to give responses of time, it was not as specific as required by the WHO coding format. For example, two mothers we interviewed gave responses of time for breastfeeding initiation as follows;

“After a while . . . ” [Mother of 4, illiterate, home delivery, Basona woreda].

“I gave birth in the morning and breastfed him in the afternoon . . .” [Mother of 2, illiterate, home delivery, Basona woreda].

We also asked mothers if they can tell the difference between minutes and hours in order to understand how accurate their responses of time were and the following extracts show how some mothers found it difficult to differentiate between minutes and hours.

MS: I am sure you heard people talk about minutes or hours, what do you understand with that and which one do you think is bigger?

“Well, when described in hours it means something has taken too much time . . . I don’t know which is bigger . . . I am illiterate. I can’t say what is what…minutes, hours etc. . . . .no . . . [Mother laughs]” [Mother of 4, illiterate, *Basona woreda*].

“I don’t know that. I only know to say 12 o’clock when it is lunch time after asking people. I don’t understand time myself” [mother of 5, illiterate, *Basona woreda*].

“well, it is the husbands who say minutes, hours etc., not me . . . and their mobile also tells time . . . [Mother laughs] . . . the mobile may not tell about minutes but it shows the hour . . . [Mother laughs]” [Mother of 3, literate, *Basona woreda*].

Mothers interviewed in this study, especially those in the rural woreda Basona, describe time in their day to day life using one or more of the following methods including by looking at sun shine shadows, cattle coming home (6 pm), asking others about the time, sun set (6 pm), husbands coming home from farming (lunch time-noon), listening to the radio to know the time, children coming from school (lunch time-noon), hearing chicken make noise (3 am) and own watch or mobile. For example, a mother of 2, illiterate and who gave birth at home in Basona woreda described how she uses the shadow from sun shine to tell the time of the day;

“We have sunshine during the day almost always. There is associated shadow that is seen in my house compound i.e. you see in the ground an area with and without shadow (sun). The shadow moves according to the intensity of the sun shine. For example, earlier in the morning, you get more shadow than the sun. As the day progress, the area covered by the sun becomes bigger and there will be less shadow. Later in the evening, the shadow increases and the sun decreases as it is getting to sun set. I have marked certain places in my house compound that correspond to the actual time of the day by monitoring the movement of the shadow and that’s how I know what time it is”.

## Discussion

Three challenges in the measurement of breastfeeding initiation have been identified in this study. Mothers’ response about the timing of early initiation of breastfeeding was related to the newborn receiving breast milk rather than their first attempt to initiate breastfeeding. In addition, we reported on how revising phrases or the wording in the early initiation question affects mother’s perception of the question. Finally, we identified difficulties among mothers in expressing time with minutes or hours which needed considerable probing.

The small sample size in this study is a limitation and the findings may not be applicable to other Ethiopian population or elsewhere in developing countries. However, it still provided an in-depth insight into how mothers could perceive the question and potential implications to accuracy in coverage reports about early initiation of breastfeeding.

Despite existing guidance, breastfeeding measurement remains a complex process and there are still variations among different breastfeeding surveys, even in how they define breastfeeding indicators [16]. For example, a recent study on breastfeeding defined early initiation as “breastfeeding initiation within the first 24 hours after birth” [17] instead of the WHO standard definition “breastfeeding within an hour after birth” [8]. DHS has two indicators for early initiation; one which measures breastfeeding within an hour after birth and a second for breastfeeding within 24 hours after birth [18]. Breastfeeding surveys rely heavily on self-reporting by mothers and in any population-based epidemiological survey self-reporting is subject to bias affecting accuracy in reported outcomes. A number of studies about dietary or energy intakes [19-22] and sexual health behavior reports [23-26] have demonstrated problems associated with self-reporting. The extent to which outcomes could be accurately reported depends on a number of factors including the length of recall period [27-30], demographic characteristics of respondents [31], inter- or intra- observer variations between data collectors [32,33], social desirability i.e. reporting about own practice based on socially acceptable norms [34-36] and approaches in data collections [34,37].

In this study, we provided evidence on how maternal responses could be influenced by how the question was asked including the wording or text description of the question i.e. approaches in data collection. WHO recommends the question (Q1 in our study) “how long after birth did you first put [name] to the breast?” in order to assess early initiation of breastfeeding [9]. However, we found out that mothers reported the time when their baby actually started receiving breastmilk rather than the time when they first put the baby to the breast. Such perceptions remained the same after we revised the question as (Q2 in our study) “how long after birth did you first put [name] in contact with you on your chest?”. The revised question (Q2), however, was mis-interpreted by some mothers due to changes in the text or wording of the question. None of the mothers we interviewed reported having skin-to-skin contact with their baby immediately after birth and there is evidence that local traditional birth attendants did not practice as such during home deliveries. Furthermore, some mothers were not even considering colostrum as part of breast milk and instead reported the time when their ‘milk came in’ or ‘burst’. This phenomenon in the process of breast milk production is technically described as lactogenesis II and some mothers do not feel increased breast fullness or the sense of ‘milk coming in’ until 72 hours after birth [38,39]. Other studies in Ethiopia also reported that even some health professionals did not consider colostrum as part of breast milk [40].

This study also identified problems related to coding maternal responses using the WHO recommended format because some mothers were unable to express time in minutes or hours. These mothers needed considerable probing in order to approximate and code their responses of time. To our knowledge, no studies have previously highlighted this concern about difficulties in expressing time by mothers. Understanding time and being able to express it in minutes, hours or days is crucial for accurate reporting about early initiation of breastfeeding. If the mother’s response is “immediately” or “less than an hour after birth”, this will be coded as early initiation of breastfeeding. If, however, her response is more than an hour after birth, then this will not be counted as early initiation of breastfeeding [9].

## Conclusion

Existing question assessing early initiation is not adequate to identify intended attempts of mothers to initiate breastfeeding. We recommend the question to be revised as “how long after birth did you first put [name] to the breast even if your breast milk did not arrive yet?”. Standard probes or follow on questions are required to avoid subjective interpretation and these should specify what early initiation is intended to measure. More guidance will also help data collectors on how to identify local socio-cultural beliefs related to breastfeeding such as colostrum avoidance and how to approximate responses of time reported by mothers who are unable to express in minutes or hours.

## Competing interests

All authors declare that they have no competing interests.

## Authors’ contributions

MS conceived and designed the study, collected and analyzed data and drafted the manuscript. SF and TM advised in the design of the study, data collection, analysis and interpretation of findings as well as critically commenting on the draft manuscript. All authors read and approved the final manuscript.

## Supplementary Material

Additional file 1 Topic guide. Click here for file
