# Supplementary material for: Measurement of breastfeeding initiation: Ethiopian mothers’ perception about survey questions assessing early initiation of breastfeeding
Source: Int Breastfeed J. 2014 Aug 25;9:13. doi: 10.1186/1746-4358-9-13 (PMC4150427; doi:10.1186/1746-4358-9-13)
Supplement: Additional file 1 — Topic guide. [file 1746-4358-9-13-S1.docx]

**Additional file 1: Topic guide for cognitive interviews**

**Questions**

Q1: “how long after birth did you first put [name] to the breast?” or

Q2: “how long after birth did you first put [name] in contact with you on your chest?”

**Probes or follow on questions**

- What is the first thing you thought up on hearing this question?
- What do you think is this question about?
- Can you repeat the question in your own words?
- Was your baby placed in contact with you on your chest soon after birth?
- Does the time you just gave me refer to when you baby actually started getting your breastmilk or the time when you attempted to initiate breastfeeding?
- How do you find having to give your responses of time as one hour, two hour or in minutes etc.?
- How do you describe time in your daily routines?
- I am sure you heard people talk about minutes and hours, which one do you think is bigger?
